# A Novel Mobile Device-Based Approach to Quantitative Mobility Measurements for Power Wheelchair Users

**DOI:** 10.3390/s21248275

**Published:** 2021-12-10

**Authors:** Jicheng Fu, Shuai Zhang, Hongwu Wang, Yan Daniel Zhao, Gang Qian

**Affiliations:** 1 Department of Computer Science, University of Central Oklahoma, Edmond, OK 73034, USA; szhang10@uco.edu (S.Z.); gqian@uco.edu (G.Q.); 2 Department of Rehabilitation Science, University of Oklahoma Health Sciences Center, Oklahoma City, OK 73104, USA; hongwu-wang@ouhsc.edu; 3 Department of Biostatistics and Epidemiology, University of Oklahoma Health Sciences Center, Oklahoma City, OK 73104, USA; daniel-zhao@ouhsc.edu

**Keywords:** bout, mobility, power wheelchair, recurrent neural network, smartphone, smartwatch

## Abstract

This study is motivated by the fact that there are currently no widely used applications available to quantitatively measure a power wheelchair user’s mobility, which is an important indicator of quality of life. To address this issue, we propose an approach that allows power wheelchair users to use their own mobile devices, e.g., a smartphone or smartwatch, to non-intrusively collect mobility data in their daily life. However, the convenience of data collection brings substantial challenges in data analysis because the data patterns associated with wheelchair maneuvers are not as strong as other activities, e.g., walking, running, etc. In addition, the built-in sensors in different mobile devices create significant heterogeneity in terms of sensitivity, noise patterns, sampling settings, etc. To address the aforementioned challenges, we developed a novel approach composed of algorithms that work collaboratively to reduce noise, identify patterns intrinsic to wheelchair maneuvers, and finalize mobility analysis by removing spikes and dips caused by abrupt maneuver changes. We conducted a series of experiments to evaluate the proposed approach. Experimental results showed that our approach could accurately determine wheelchair maneuvers regardless of the models and placements of the mobile devices.

## 1. Introduction

There are more than 200,000 power wheelchair users in the United States [1]. Meanwhile, the demand for power wheelchairs increases substantially every year [2]. As the aging population continues to grow, power wheelchairs will play an increasingly important role in helping individuals maintain independent mobility.

Mobility is an important indicator of quality of life and is determined by the World Health Organization (WHO) as the key component of activity and social participation [3,4]. However, research has shown that people with disabilities are more likely to live a sedentary lifestyle, which has been proven to be harmful and associated with various chronic diseases, such as cardiovascular disease, diabetes, obesity, etc. [5,6,7]. It is therefore important to quantitatively measure whether a power wheelchair user remains active and therefore lives a healthy lifestyle. Among the mobility metrics, the mobility bout, which represents a period of continuous wheelchair movement, is particularly useful in measuring activity and participation because a bout denotes a transition between locations [4]. Hence, the number of bouts can reveal activities performed at different locations by a power wheelchair user [4]. In this study, we consider bouts as the primary measurement of mobility, based on which we can determine several critical mobility metrics, such as the accumulated movement time and maximum and average periods of continuous movement [3,4,8].

Unfortunately, despite the advancement of technology and great efforts spent on assistive technology, approaches that support quantitative mobility assessment are still not readily available in the daily lives of power wheelchair users [9]. Existing approaches typically attach sensors to the wheels of a wheelchair to collect wheelchair maneuvering data [10,11,12]. In [4,13], a reed switch was mounted to the wheelchair frame with two to four magnets evenly attached to the wheel. The wheel rotations were counted as when the magnets crossed the reed switch. In a recent study [14], researchers used a Bluetooth-based wheel rotation monitor, which consisted of a sensor and a magnet attached to a manual wheelchair’s axle and spokes of a wheel, respectively, to collect wheelchair maneuvering data. Research participants also wore a smartwatch to collect accelerometer data caused by hand activities (e.g., propulsion). The wheel rotation monitor and the smartwatch were connected with a smartphone for near real-time data transmission. Researchers in [10,15] further simplified the instrumentation by directly mounting an accelerometer to the spokes of a wheelchair. When the wheel rotated, the accelerometer data demonstrated a strong pattern resembling sinusoid-like curves, making data analysis much easier. Unfortunately, such approaches can hardly be used in daily life. First, it is impractical to request a power wheelchair user with mobility impairment to install the sensors on the wheels. Second, the maintenance of these sensors, e.g., periodically recharging and reinstalling the sensors, offloading data into inventory, etc., requires specialized expertise. These factors create substantial challenges and extra burden for power wheelchair users to adopt these approaches.

In our study, we aim to develop a mobile-device-based approach that does not rely on additional sensors for data collection. Instead, wheelchair users can simply use their own mobile devices, e.g., a smartphone or smartwatch, to collect mobility data. A recent related study [16] proposed to use a smartphone attached to the armrest of a manual wheelchair to collect accelerometer data for wheelchair activity analysis. Our approach differs in the following three aspects: (1) our focus is on power wheelchairs, which have different dynamics compared to those of manual wheelchairs; (2) the study in [16] used a deep convolutional neural network (CNN) for data analysis, while our approach uses a deep recurrent neural network (RNN) [17], which is effective in processing temporal data; and (3) the CNN in [16] was designed to classify whether the wheelchair was operating on an indoor flat surface or outdoor grasslands, while ours was to measure mobility.

Prevalent health tracking apps are also related to our work. These apps are designed for individuals without physical impairments, where measurements are achieved through detecting a person’s steps and body oscillation [18]. However, the dynamics of a power wheelchair do not possess the same characteristics of steps or body oscillation. Instead, the acceleration and deceleration periods of a power wheelchair are instantaneous. Such rapid changes in wheelchair maneuvers can be easily overwhelmed by noise, thus making it difficult to determine a wheelchair’s moving status. Furthermore, data collected by inertial sensors in a smartphone demonstrate a wide variety of differences. The patterns of sensor data may vary from building to building, let alone the significant differences between indoor and outdoor settings. In addition, there are many mobile device manufacturers that produce a large number of different models. The challenge is that the built-in sensors in different mobile devices could create significant heterogeneity in terms of sensitivity, noise patterns, sampling settings, etc. It is therefore desirable to develop an approach that can accurately determine a wheelchair’s maneuvering status regardless of the specific mobile device used for data collection.

To address the aforementioned challenges, we developed a novel approach composed of algorithms for noise reduction, wheelchair maneuver determination, and data post-processing. Specifically, we proposed a new dynamic differential reference algorithm (DDR) to mitigate the impact of noise. The DDR algorithm transforms raw sensor data through value differentiation. The transformed data preserves patterns intrinsic to wheelchair maneuvers, i.e., the data of stationary periods shows a uniform characteristic, while data of moving periods demonstrates distinct patterns. Then, we employed cutting-edge machine-learning techniques to recognize such patterns to determine mobility bouts to analyze a power wheelchair user’s mobility. The use of machine-learning techniques also makes our approach more robust to the noise that is left over by DDR. Specifically, we developed an RNN [17], which is particularly suitable for this study because RNNs excel in processing temporal data series, such as the sensor data collected over a time period. Since RNNs are sensitive to maneuver changes, e.g., sudden accelerations or decelerations, their outputs may contain spikes or dips that may divide a bout into multiple smaller ones. We developed a post-processing algorithm to smooth the outputs of the RNN so that the final output can accurately reflect the true maneuvering status of a power wheelchair.

We conducted a series of experiments to evaluate the proposed approach. We used eight smart mobile devices of different manufacturers and models, including seven smartphones and one smartwatch, to collect wheelchair maneuvering data. The experiments were conducted inside an academic building that had different terrains, e.g., upward/downward slopes, curving and flat floors, etc. Experimental results showed that our proposed approach could accurately determine mobility bouts no matter which mobile devices were used. The outputs from our approach matched very well with the ground truth (e.g., by manually recording bout durations or by dedicated sensors attached to the driving wheels of a power wheelchair to record the wheelchair’s maneuvering status). The outputs from the eight mobile devices were also highly correlated, indicating consistent analysis results over these heterogeneous devices. Hence, the experimental results suggest that our proposed approach provides a general solution independent of specific mobile devices for mobility analysis.

The rest of the paper is organized as follows. In Section 2, we present our algorithms for data preprocessing, the construction of RNN pattern recognition model, and data post-processing as well as experimental design. In Section 3 and Section 4, we show and discuss the experimental results. In Section 5, we conclude the paper and identify future research directions.

## 2. Materials and Methods

We developed an Android app that can capture sensor readings from the built-in accelerometer of a mobile device. An accelerometer can measure accelerations along three axes of the mobile device. To configure the frequency of data sampling, Android APIs provided four data sampling settings for accelerometers, i.e., SENSOR_DELAY_FASTEST, SENSOR_DELAY_GAME, SENSOR_DELAY_UI, and SENSOR_DELAY_NORMAL in the order from high sampling frequency to low [19]. Each setting corresponded to a range of data sampling rates instead of a fixed one. The reason for this is that the built-in accelerometer was event-driven, i.e., it output a sensor reading whenever it detected a speed change. Correspondingly, we also recorded the time stamp with microsecond precision for each sensor reading to facilitate data analysis. Therefore, the sequence of raw data collected by an accelerometer can be modeled as a matrix:

(1) $$M\in {\mathbb{R}}^{n\times 4}$$

where *n* is the number of raw sensor readings collected over a certain time period, and each row is a 4-tuple, i.e., *R_i_* = {*a^x^_i_*, *a^y^_i_*, *a^z^_i_*, *t_i_*} ∈ ${\mathbb{R}}^{1\times 4}$, which consists of 4 items, namely the accelerations along three axes, *x*, *y*, and *z*, and the time *t_i_* when the sensor reading is recorded.

### 2.1. Data Preprocessing

An accelerometer is subject to both sensor and environmental noise, which may vary greatly when a wheelchair is moving in different environmental settings. To mitigate the impact of noise, we developed a new dynamic differential reference algorithm (DDR). The DDR algorithm takes four steps to process raw accelerometer data *M*.

#### 2.1.1. Noise Reduction

First, DDR calculates the differences between consecutive sensor readings along a specific axis, e.g., *x*, *y*, or *z*. Specifically, in an axis, a sensor reading can be denoted as:

*α* = *α_T_* + *α_N_* (2)

where *α* is the sensor reading, *α_T_* is the true acceleration, and *α_N_* is noise. Based on our empirical observations, noise *α_N_* tends to have similar values within a very short period of time. Hence, the differentiation of two consecutive sensor readings, *α*_1_ = *α*_1*T*_ + *α*_1*N*_ and *α*_2_ = *α*_2*T*_ + *α*_2*N*_, will roughly remove noise, i.e., *α*_2_ − *α*_1_ = (*α*_2*T*_ − *α*_1*T*_) + (*α*_2*N*_ − *α*_1*N*_) ≈ *α*_2*T*_ − *α*_1*T*_.

Table 1 illustrates an example of value differentiation along the *y*-axis. The columns “Stationary (Raw)” and “Moving (Raw)” contain two data segments corresponding to the stationary and moving status, respectively. Similarly, the columns of “Stationary (Differentiated)” and “Moving (Differentiated)” show the data differentiated from the corresponding raw data segments. The raw data show that even though the wheelchair is stationary, the sensor readings still have much larger values than the expected 0. The distinction between the stationary and moving raw data is also not very clear. As a result, it will be difficult to analyze wheelchair maneuvering status based on the raw data. In comparison, the differentiated values show distinct patterns between stationary and moving periods as the values in the stationary data segment are close to 0, while the absolute values in the moving data segment are larger than 0.

After applying the DDR algorithm to all three dimensions, we obtain a new differentiated matrix:

*M*′ = {*R_x_*′, *R_y_*′, *R_z_*′, *T*} (3)

where *R_x_*′, *R_y_*′, and *R_z_*′ are column vectors along the *x*, *y*, and *z* axes, and *T* is the column vector of time indicating when each sensor reading is recorded.

#### 2.1.2. Combining Sensor Data along the Three Axes into a Single Sequence

As the second step, DDR combines *R_x_*′, *R_y_*′, and *R_z_*′ as defined in Equation (3) into a single sequence *D* to minimize the influence of differences in device placement, i.e., making it more placement-invariant.

(4) $$M^{\prime \prime }=\text{\{}(d_{i}\;,\;t_{i})\;|\;t_{i}\in T\mathrm{and}d_{i}=\sqrt{a_{x}^{i{}^{\prime }}{}^{2}+a_{y}^{i{}^{\prime }}{}^{2}+a_{z}^{i{}^{\prime }}{}^{2}}\in D\mathrm{with}{a^{i{}^{\prime }}}_{x}\in {R_{x}}^{\prime },\;{a^{i{}^{\prime }}}_{y}\in \;{R_{y}}^{\prime },\mathrm{and}{a^{i{}^{\prime }}}_{z}\in {R_{z}}^{\prime }\text{\}}$$

#### 2.1.3. Outlier Processing

**Figure 1:**
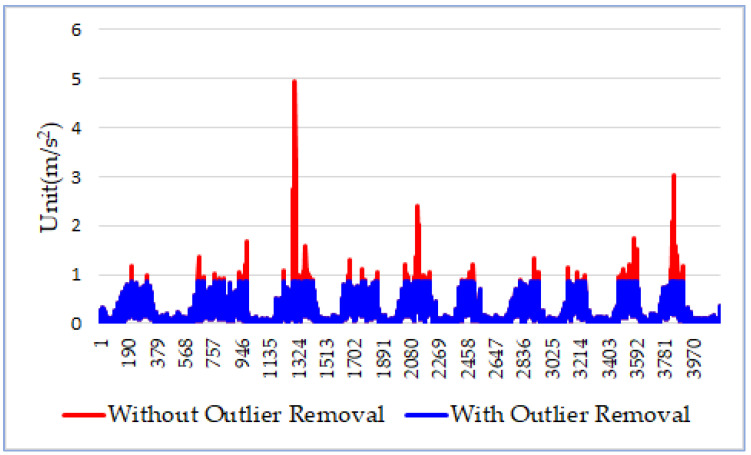
Outlier removal.

In the third step, DDR removes outliers from data sequence *D* (as shown in Equation (4)) to ensure that the true patterns associated with wheelchair movements will not be obscured. To identify outliers, we use the quartiles and interquartile range (IQR) method [10], in which three quartiles (i.e., *Q*_1_, *Q*_2_, and *Q*_3_) divide the ordered data set into four equal sections. In statistics, the outliers fall outside the range of [*Q*_1_ − 1.5 × IQR, *Q*_3_ + 1.5 × IQR], where IQR = *Q*_3_ − *Q*_1_. In our case, the valid data range is [0, *Q*_3_ + 1.5 × IQR] because all data items in *D* (Equation (4)) have values greater than or equal to 0. Hence, we simply replace outliers with *Q*_3_ + 1.5 × IQR, which is the upper bound of the range. As shown in Figure 1, the red curve is the data sequence *D* containing outliers, which are significantly larger than the regular sensor values. The blue curve represents the data sequence with outliers rescaled. The horizontal axis represents the order of the data items in the sequence *D*, and the vertical axis represents the differentiated accelerations.

#### 2.1.4. Data Normalization

In the last step, DDR employs the MinMax scaler [20] to normalize sensor values into the range [0, 1]. This feature-scaling step is critical because it makes sensor data from any mobile devices fall into the same range. We denote the normalized data sequence as *D_s_*:

*D*_*s*_ = *MinMax*(*D*) (5)

**Figure 2:**
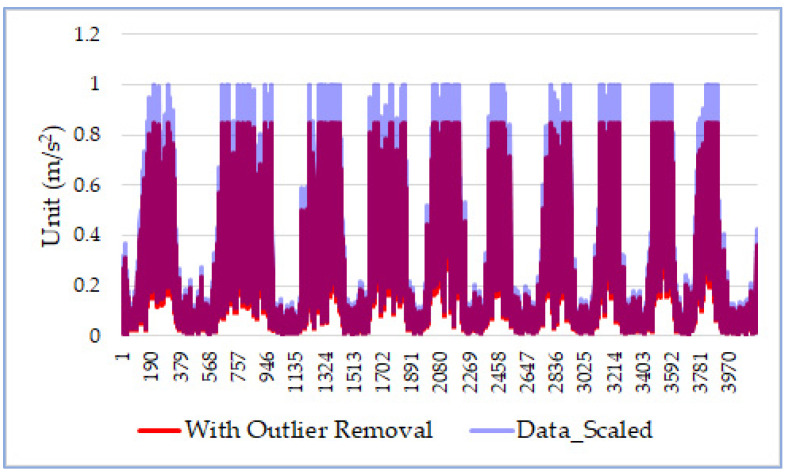
Data scaling.

As shown in Figure 2, after removing outliers, the data sequence (i.e., the red curve) has an upper bound smaller than 1. The feature-scaling step normalizes the data sequence to the range between 0 and 1. As in Figure 1, the horizontal axis represents the order of the data items in the sequence *D_s_*, and the vertical axis represents the differentiated accelerations.

### 2.2. Machine Learning Models

**Figure 3:**
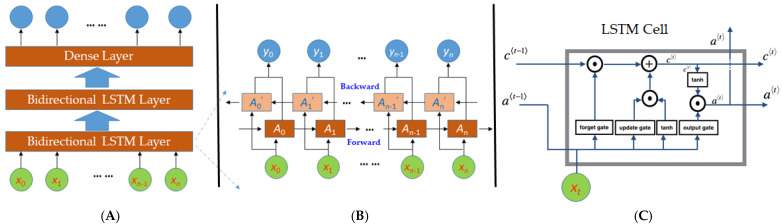
Neural network structure. (**A**) Overall network structure, (**B**) A bidirectional LSTM Layer, (**C**) An LSTM Cell.

In this study, we used the recurrent neural network (RNN) [17] to train models that can determine a wheelchair’s maneuvering status. The training was based on the dataset *D_s_* defined in Equation (5). We chose an RNN because it excels at handling temporal data sequences. As shown in Figure 3A, our RNN consists of an input layer, two bidirectional LSTM (long short-term memory) layers [21], a dense layer (i.e., a fully connected layer) [22], and an output layer.

The input layer is composed of a series of data segments, *x_i_* (0 ≤ *i* ≤ *n*), i.e., the data sequence *D_s_* defined in Equation (5) is evenly divided into smaller data segments *x_i_*. The size of a data segment *x_i_* should be neither too small nor too large. If the size is too small, *x_i_* contains too little information for the LSTM unit to analyze. On the other hand, if the size is too big, the data segment may span across multiple stationary and moving maneuvers. In this study, we chose the size to be 10, which is the amount of data collected within 1 s for most of the mobile devices. For example, the sampling rates are between 13 to 30 Hz at the predefined Android sampling setting SENSOR_DELAY_UI for the mobile devices used in our experiments. This empirical setting is useful to preserve precision when a data segment *x_i_* represents a transition from one maneuver to another, i.e., it contains data of both maneuvers. No matter how RNN classifies this data segment, i.e., stationary or moving, only a portion of the 10 data items is misclassified, i.e., the lost precision is less than 1 s.

A bidirectional LSTM layer contains two hidden layers, i.e., a forward and a backward LSTM layer, as shown in Figure 3B. The choice of the bidirectional LSTM layer was inspired by our manual procedure of analyzing the maneuvering status at a specific data point. We typically needed to inspect the data series before and after this particular point to determine the true moving status. Similarly, the bidirectional LSTM layers provide comprehensive information, including both forward and backward maneuvering information, to the next layer in the network. In an LSTM layer, the LSTM cells are the basic units, which are connected into a sequence. The LSTM cells are responsible for keeping track of the maneuvering status. At any point in time, an LSTM cell needs to decide whether to keep or update the maneuvering status passed from its preceding LSTM cell. This decision is made by the collaboration of three gates, i.e., the forget, update, and output gates, as shown in Figure 3C. Here, *c*^〈*t*−1〉^ and *a*^〈*t*−1〉^ are the cell status and cell value from the preceding LSTM, respectively. If the maneuvering status of a wheelchair changes from moving to stationary or vice versa at time *t*, the forget gate will produce an output to nullify *c*^〈*t*−1〉^. Here, the symbol of a circle with a dot means element-wise matrix multiplication. Hence, if an item in the matrix produced by the forget gate is 0, it means that the LSTM cell will forget the corresponding value in *c*^〈*t*−1〉^. In the case that the cell has to forget *c*^〈*t*−1〉^, the update gate will make sure that a new cell status is updated to reflect the new status. The symbol of a circle with a plus means matrix addition that retains values from all input matrices. Similarly, the output gate determines the output to the next LSTM cell and layer.

The dense layer is a fully connected layer consisting of 20 neurons. The dense layer is responsible for high-level reasoning that facilitates the decision-making in the output layer. The output layer is a sigmoid layer [23] that generates a series of 0 s and 1 s, where 0 represents stationary and 1 represents moving. It is worth mentioning that the network structure, including the number of layers and the number of neurons in each dense layer, are hyperparameters [24], which define the RNN model, but cannot be determined through training. The common practice is to manually tune these parameters through experiments. In this study, we determined the network structure through experiments. In fact, we did not spend too much time on the selection of the optimal network structure because our DDR algorithm can preserve the inherent patterns of wheelchair maneuvers, which makes the subsequent pattern recognition much easier. As discussed in Section 4, all the tested networks demonstrated reasonable accuracy with the proposed RNN achieving the best result.

### 2.3. Data Post-Processing

**Figure 4:**
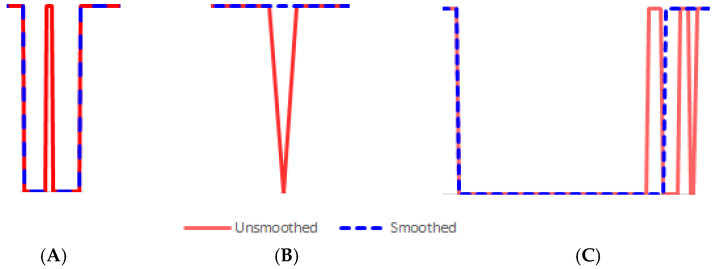
Curve smoothing. (**A**) A spike, (**B**) A dip, (**C**) An unsmooth maneuver.

Although the proposed RNN can correctly classify the sequential data, the output curve may not be smooth due to abrupt maneuver changes, e.g., sudden acceleration/deceleration, start/stop, etc. Therefore, data post-processing is necessary to smooth the data curve for more accurate bout recognition. Our post-processing algorithm mainly handles three situations, as shown in Figure 4. The first situation represents a spike caused by an unexpected external force when the wheelchair is stationary, as shown in Figure 4A. The red curve with a spike is the output from the RNN, while the dashed blue curve is the output from the proposed post-processing algorithm, which eliminates the spike by merging it into the adjacent data segments. Figure 4B shows a dip (the red curve) that occurs when the wheelchair is moving while experiencing a big change in speed. The post-processing algorithm simply merges the dip into the adjacent data segments. The third situation happens when the maneuver of a power wheelchair is not smooth, e.g., turning, sudden starts/stops, etc. As shown in Figure 4C, the red curve represents the output from the RNN. The oscillation happens when the wheelchair begins to move. Our post-processing algorithm splits the oscillation period into two parts, i.e., 2/3 is assigned to the moving period and 1/3 to the stationary period. Such a split is based on the rationale that the wheelchair is mostly moving over the oscillation period.

### 2.4. Experiments

**Figure 5:**
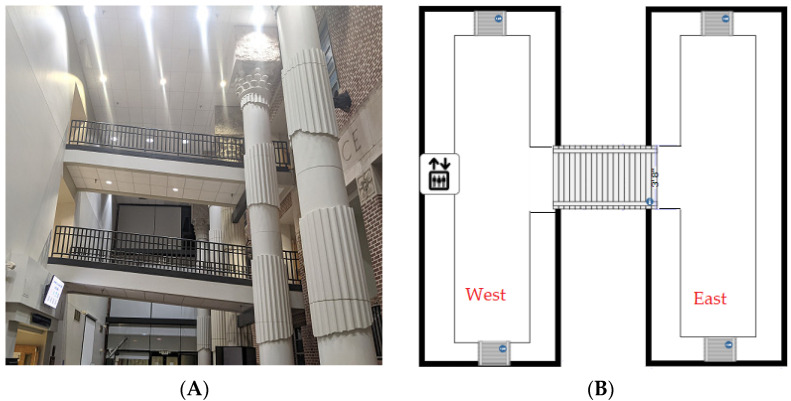
Experimental setting. (**A**) A side view of the overall building, (**B**) A bird’s eye view.

We conducted a series of experiments inside an academic building. As shown in Figure 5A, the building’s east and west wings are connected by sloped bridges at the second and third floors. Figure 5B illustrates the bird’s eye view of the building. This experimental setting is ideal in testing our proposed approach because it contains various terrains, such as flat floor, slopes, interconnections, and bumps, to simulate different scenarios in real life.

#### 2.4.1. Experimental Series 1

**Figure 6:**
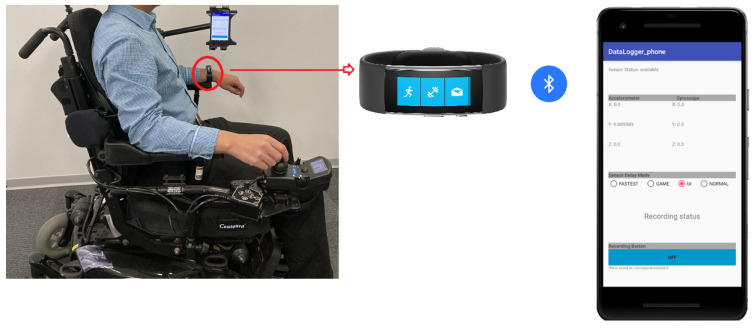
System settings for experimental series 1.

We conducted the first series of experiments (6 trials) on the second floor by using an LG G2 (D800) smartphone and a smartwatch (Microsoft Band 2) to collect wheelchair maneuvering data. For both devices, we set the sampling frequency to be SENSOR_DELAY_UI, which could achieve satisfactory precision while consuming less battery power of the smart mobile device, as determined in our previous study [15]. As shown in Figure 6, the smartphone was placed into a holder (Arkon Tab-802) attached to a power wheelchair (Invacare FDX). The driver wore the watch on his left hand while using his right hand to manipulate the joystick of the wheelchair. His left hand remained still during the experiments. The smartwatch communicated with the phone app through Bluetooth.

The itinerary of each trial always started from the west wing of the building, driving through the bridge, either turning to left or right at the end of the bridge into the east wing, making a U-turn at the end of the corridor, and then driving back to the original start point. We preset the waypoints to remind the wheelchair user where to stop or turn. During the experiments, researchers also recorded the durations of the bouts using a timer, which served as the ground truth to validate our proposed approach.

We used the phone data from the first trial (Trial 1) to train our RNN model. The phone data from the second trial (Trial 2) was used for validation and testing, in order to avoid overfitting, a situation where the trained RNN overly fits the training data set, thus making it unable to generalize to classify unseen data. Specifically, data from the second trial was split into 25% (validation set) and 75% (testing set). The early-stopping technique [25] was used during training when the classification accuracy over the validation set did not improve within 100 epochs. Once the RNN model was trained, we used it to analyze data from the remaining four trials (Trials 3–6), including both phone and smartwatch data.

#### 2.4.2. Experimental Series 2

**Figure 7:**
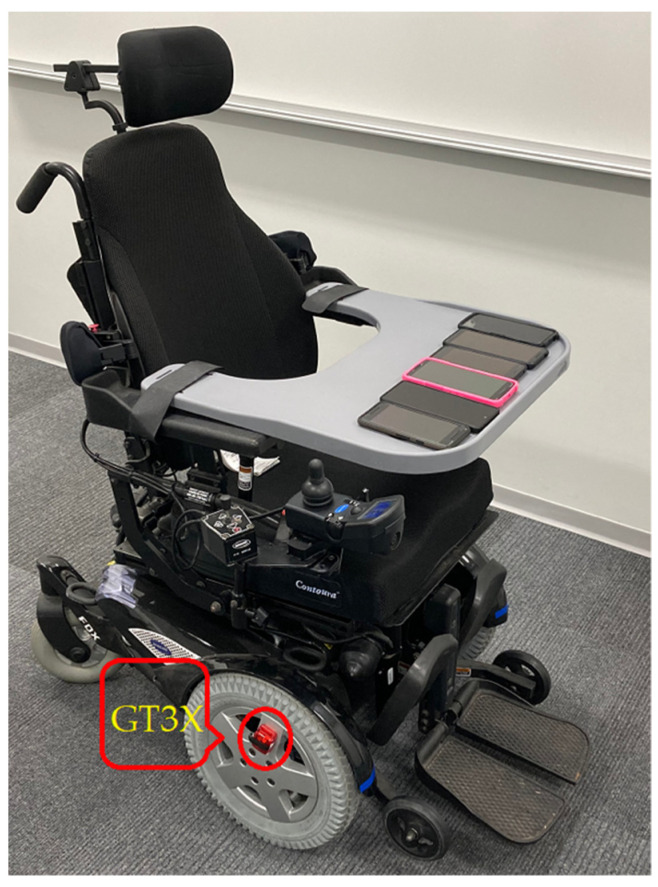
System settings for experimental series 2.

Since our manual recording of bout durations could be subjective and error-prone, we conducted a second series of experiments, in which we obtained the ground truth by using the approach of existing research [10,15], i.e., placing a dedicated high-precision accelerometer (ActiGraph GT3X [26]) to both driving wheels of the power wheelchair, as shown in Figure 7. Besides obtaining the ground truth, we can also compare our proposed approach with the existing approach in mobility measurement. In addition, as shown in Section 3, the outcomes from the phone and smartwatch were highly correlated. Accordingly, we hypothesized that our proposed approach should work well with other types of Android phones as well. To verify this hypothesis, in the second experiment, we used six different Android phones, i.e., Google Pixel, Pixel 2, Pixel 3, Motorola Nexus 6, Samsung A71, and ZTE Axon. All the phones were placed on a tray instead of the smartphone holder. The purpose was to examine whether the RNN model, trained by using data collected by placing the phone into a smartphone holder, could correctly classify data collected from a totally different setting. This experimental series was conducted on the third floor of the academic building, which was another difference from the first experimental series, which was performed on the second floor. Two divers participated in the experiments. Each driver conducted four trials, two with the first gear and two with the second gear of the power wheelchair. Each trial contained two bouts. The first bout started from the west wing, passed through the bridge, turned left into the east wing, and stopped at the end of the corridor. The second bout followed the same route driving back from the east wing to the west wing.

All the trials in both experimental series include typical maneuvers, such as accelerations, decelerations, linear maneuvers, left turns, right turns, and spot turns. Spot turns refer to the rotation of a wheelchair without changing its location. In experimental series 2, we also tested our application at different maneuvering speeds. Invacare FDX provides four Drive options, with the top speed being 5 miles/hour. Drive 1 (INDR_AVG) is for indoor operations; Drive 2 (MOD_OUTDR) is for outdoor operations with low to moderate speeds; Drive 3 (SPEED_LVL) is for full-speed operation; Drive 4 (RAMP_CURB) is for outdoor driving on the ramp or curb. In experimental series 2, we used Drive 1 and Drive 2 to achieve varying wheelchair speeds that were safe and comfortable for normal daily use in indoor settings.

## 3. Results

**Figure 8:**
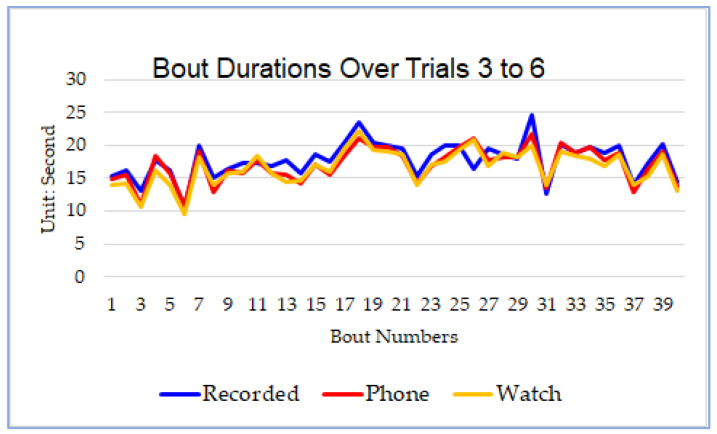
Bout durations over Trials 3 to 6.

Figure 8 illustrates the results for the first experimental series. The horizontal axis shows the bout number, while the vertical axis shows bout durations measured in seconds. The blue curve (i.e., recorded) represents the ground truth obtained by using a timer to record bout durations. The curves fit very well in most of the bouts. In general, the bout durations determined by our proposed approach were a little shorter than the recorded ones. The bout durations obtained based on the data from the smartwatch were slightly shorter than those from the phone.

Table 2 shows the more detailed experimental results. The “Recorded” column contains the ground truth. On average, the difference between the phone-based results and the recorded bout durations is 1.16 s (6.62%), and the difference between the watch-based results and the recorded bout durations is 1.52 s (8.6%). In addition, we also analyzed the pairwise correlations among the recorded, phone-based, and watch-based results. As shown in Table 3, all three data series of bout durations are highly correlated. Particularly, the phone-based and watch-based results have a higher correlation, 0.95, which supports our hypothesis that our approach should work well with different kinds of devices regardless of their placement.

Table 4 shows the results from the second experimental series. The “GT3X” column represents the ground truth for bout duration comparisons. The average difference against the GT3X results falls into the range of [2.22%, 5.27%]. Hence, our proposed approach achieved very good accuracy on the data from all six phones. The averaged standard deviation is 2.03 s in an averaged bout duration of 53.56 s. In addition, we evaluated the pairwise correlations among all the devices. As shown in Table 5, bout durations based on different devices are highly correlated.

## 4. Discussion

In this study, we demonstrated the feasibility of building an effective model that can accurately determine a power wheelchair’s maneuvering status on top of heterogenous mobile devices. Specifically, we developed an RNN based on the data collected using an LG G2 (D800) smartphone. Then, we used this RNN to classify wheelchair maneuvering data collected by a smartwatch (MS Band 2) and six other smartphones, i.e., Google Pixel, Pixel 2, Pixel 3, Motorola Nexus 6, Samsung A71, and ZTE Axon. The classification results were then further smoothed by our post-processing algorithm to remove spikes and dips. Experimental results showed high correlations (as shown in Table 3 and Table 5) among all mobile devices in bout determination.

It is also worth noting that the training data were obtained on the second floor of the academic building when the LG G2 smartphone was attached in a smartphone holder. The testing data sets were collected on the second floor when a research participant wore the smartwatch on the wrist, and on the third floor when the six other smartphones were placed on a wheelchair tray. In other words, the mobile devices used in this study were positioned very differently during data collection, while the indoor settings and wheelchair users were also different. This demonstrates the effectiveness of the proposed DDR algorithm in removing noise and eliminating the impacts caused by differences in wheelchair users and the placement of the mobile devices. Hence, the DDR algorithm can preserve the essential patterns of various wheelchair maneuvers. As a result, the proposed RNN can recognize these patterns no matter how a smart mobile device is positioned. Our study proves that it is possible to eliminate the influence from the large variations of mobile devices, e.g., the placement of a smart mobile device, its sampling rate, the scales of its sensor data, its sensor sensitivity, etc., by capturing the essential patterns intrinsic to wheelchair maneuvers.

Further, the results obtained by using our approach were also close to the ground truth. As discussed in Section 2, we used two approaches to obtain the ground truth, i.e., manually recorded bout durations using a timer in experimental series 1 and by attaching GT3X accelerometers to both driving wheels of the power wheelchair in experimental series 2. Compared to the ground truth in experimental series 1, the average difference was only 1.16 s (6.62%) for phone-based results and 1.52 s (8.60%) for watch-based results, as shown in Table 2. Similarly, in experimental series 2, the average differences between the phone-based results and the ground truth varied from 1.15 s (2.22%) to 2.75 s (5.27%) among the six phones, as shown in Table 4. As existing research typically attaches dedicated sensors to the wheels of a wheelchair to measure mobility [10,11,12], the ground truth obtained from GT3X in experimental series 2 also allows us to compare our proposed approach with existing methods. As shown in Table 5, the pairwise correlation analysis demonstrates high correlations between GT3X and six phones, which suggests that our approach has achieved comparable accuracy with existing methods.

If we compare the results of two experiments series in Table 2 and Table 4, it can be seen that the percentage of differences in experimental series 2 is relatively lower. The reason for this is that when using our approach to determine bout durations, the major differences against ground truth lie in the start and end points of a bout. This is understandable because even researchers find it very challenging to manually determine the exact start and end points of a bout by looking through the sensor data series. However, once a wheelchair moves steadily, the data patterns become clear so that our RNN can accurately determine its maneuvering status. Because the bout durations in experimental series 2 were much longer than those in experimental series 1, the variations at the start and end points only accounted for a small portion of the entire bout duration, thus resulting in a lower difference percentage. In general, the longer a wheelchair moves continuously, the more accurately our proposed approach can determine the bout duration. This will be very important for the estimation of cumulative activities in real-life cases.

**Figure 9:**
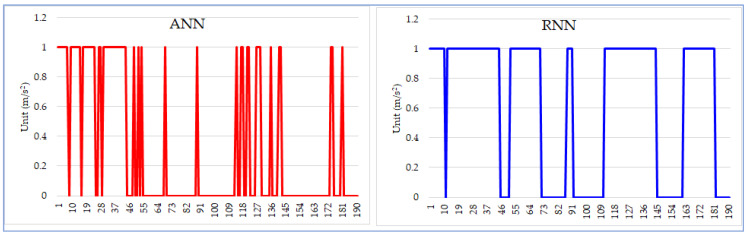
ANN vs. RNN.

In addition to the RNN, we also investigated the applicability of the traditional artificial neural network (ANN) [27] to classify wheelchair maneuvering data. We trained an ANN using the same training and testing data sets as we did with our RNN. We found that if a wheelchair moves regularly, the differences between ANN and RNN were not significant. However, if a wheelchair experiences unexpected bumps, the ANN may have difficulty in determining the wheelchair’s moving status because its classification decision is solely based on the current input. In contrast, a bidirectional RNN makes a decision based on not only the current input but also the status denoted by its prior and subsequent data segments. For example, the data in Figure 9 was obtained during an experiment when the wheelchair accidentally scratched the wall during a turn and the research participant tried to drive the wheelchair back to the normal route. As shown in Figure 9, the classification results from the ANN are full of dips and spikes. In comparison, the outcomes from the RNN are much smoother and more accurate. As a result, the subsequent post-processing algorithm can easily remove dips and spikes to further smooth the curve.

Some GPS-based approaches were proposed to assess an individual’s mobility [28,29]. While such approaches can accurately measure mobility, their usability is limited to outdoor activities as GPS signals are mostly unavailable indoors. Our proposed approach is more general since it can be used in both indoor and outdoor settings. In addition, it can work with GPS-based approaches to provide a more comprehensive and effective solution to assess power wheelchair users’ mobility in their daily lives.

It is quite feasible to extend our mobile device-based approach to a cloud computing setting. The integration of mobile and cloud computing can potentially yield substantial clinical benefits. Healthcare professionals will be able to take care of more patients remotely and stay focused on those who demonstrate sedentary lifestyles in their daily life.

### Study Limitations

The smartphones used in this study were all Android phones, which have the highest market share (72.19%) [30]. In the next step of our research, we will investigate iPhones and Apple watches that run on iOS operating systems. In addition, the mobile devices were only used for data collection during experiments. In other words, we did not take phones off the smartphone holder or pick up phones from the tray for other activities, although such activities are rare while the wheelchair is moving. The hand that wore the smartwatch was not used for driving and remained in the same place throughout the experiments. However, in everyday life, the wheelchair user may use the phone for texting, calling, surfing the Internet, etc. Such non-wheelchair-related activities may be mis-classified as wheelchair maneuvers. As a next step, we will provide more fine-grained classifications to distinguish wheelchair- and non-wheelchair-related activities to segment the meaningful periods for mobility analysis.

Our study lays a solid foundation towards a generic approach to quantitative mobility measurement that is independent of mobile devices and power wheelchairs used in daily life. The results of the current study were obtained based on one type of power wheelchair (Invacare FDX). In the next step, we will further validate the proposed approach using wheelchairs from different manufacturers and with different mechanical configurations, e.g., front-wheel, rear-wheel, or mid-wheel drive.

## 5. Conclusions

In this study, we presented an approach for analyzing power wheelchair users’ mobility by using accelerometer data from a smartphone/smartwatch. As there are a great number of such mobile devices available, our goal was to make our proposed approach independent of any specific mobile device used for data collection. Our approach consisted of a data preprocessing algorithm, DDR, an RNN, and a postprocessing algorithm. The DDR algorithm helped remove noise, outliers, and normalize sensor data. The processed data demonstrated strong patterns associated with wheelchair maneuvers. Then, the RNN was responsible for recognizing these patterns to determine a wheelchair’s maneuvering status. Finally, the postprocessing algorithm smoothed the outputs from the RNN to remove unnecessary spikes and dips. We conducted two series of experiments to evaluate the proposed approach. In experimental series 1, we used a smartphone (attached to a holder) and a smartwatch to collect wheelchair maneuvering data. The ground truth was obtained by manually recording bout durations using a timer. In experimental series 2, we used six different Android phones that were placed on a wheelchair tray to collect the data. The ground truth was collected by attaching GT3X accelerometers to both driving wheels of a power wheelchair. Experimental results have confirmed that our approach can handle variations in data collected from different mobile devices by capturing the patterns intrinsic to wheelchair maneuvers. Specifically, our approach achieved high accuracy across all the mobile devices compared to the ground truth. Second, the analysis results obtained from the mobile devices were highly correlated. This shows good potential for our proposed approach to be extended to real-life activities. Additionally, by the nature of this quantitative mobility study, no special expertise is needed from wheelchair users. Hence, its non-intrusive features with minimum extra cost could also be contributing factors to its further adoption. Therefore, our approach laid the groundwork for a service-ready solution that can be easily integrated into a power wheelchair user’s daily life to promote healthy behavior changes.

In the next step, we will extend our study to iOS devices so that all mainstream mobile devices will be supported. We will also make our approach capable of providing more fine-grained analysis by distinguishing wheelchair- and non-wheelchair-related activities. Advanced hyperparameter tuning approaches will be employed to refine the neural network structure to further improve its accuracy.

## 6. Patents

Jicheng Fu and M. Haff, Intelligent apparatus for patient guidance and data capture during physical therapy and wheelchair usage, US Patent No. 10,182,766, 2019 and US Patent No. 1,033,238, 2021.

## Figures and Tables

**Table 1:** Raw vs. differentiated data (unit: m/s^2^).

Stationary (Raw)	Moving (Raw)	Stationary (Differentiated)	Moving (Differentiated)
3.405087	3.432328	0.01362	−0.49033
3.364226	2.941995	0.027241	0.39499
3.350606	3.336985	0.040861	0.231546
3.309745	3.568531	−0.04086	0.463092
3.350606	4.031623	−0.01362	0.177064
3.350606	4.208687	−0.04086	−0.40861
3.418707	3.800077	0.040861	−0.27241
3.364226	3.52767	0	−1.19859
3.336985	2.329079	0.068102	2.955616
3.350606	5.284695	−0.05448	−2.50614

**Table 2:** Results for the first experimental series (unit: s).

	Trial 3			Trial 4			Trial 5			Trial 6		
	Recorded	Phone	Watch	Recorded	Phone	Watch	Recorded	Phone	Watch	Recorded	Phone	Watch
**Bout1**	15.33	14.97	14.00	17.32	17.77	18.41	19.55	18.49	18.60	12.7	13.52	13.90
**Bout2**	16.3	15.57	14.15	16.88	15.68	15.69	15.32	14.20	13.97	20.15	20.31	19.01
**Bout3**	13.07	10.80	10.77	17.76	15.63	14.36	18.63	16.93	17.05	18.95	18.96	18.34
**Bout4**	17.65	18.35	16.30	15.85	14.26	14.58	19.86	18.28	17.53	19.83	19.66	17.97
**Bout5**	16.23	15.69	14.07	18.61	16.99	17.11	19.92	19.71	19.23	18.83	17.73	16.96
**Bout6**	10.5	10.87	9.64	17.57	15.56	15.93	16.52	20.98	20.84	19.9	18.95	18.66
**Bout7**	19.88	19.17	18.23	20.33	18.35	19.56	19.49	17.74	16.93	14.03	12.98	14.06
**Bout8**	15.13	12.91	14.00	23.58	21.07	22.08	18.73	18.29	18.85	17.4	16.37	15.41
**Bout9**	16.31	16.30	15.74	20.43	19.63	19.32	18	18.29	18.09	20.26	19.16	18.53
**Bout10**	17.35	15.69	15.95	19.93	19.78	19.02	24.56	21.68	20.02	14.41	13.72	13.01
				Phone				Watch				
**Average Difference against the Recorded**				1.16 s (6.62%)				1.52 s (8.60%)				

**Table 3:** Pairwise correlation for the first experimental series.

	Recorded	Phone	Watch
**Recorded**	1.00	0.90	0.88
**Phone**	0.90	1.00	0.95
**Watch**	0.88	0.95	1.00

**Table 4:** Results for the second experimental series (unit: s).

	GT3X	Nexus 6	Pixel-1	Pixel-2	Pixel-3	Samsung	ZTE	Standard Deviation
**Bout1_1**	52.33	52.00	50.69	51.16	51.28	50.61	49.88	0.72
**Bout1_2**	64.08	61.65	52.25	57.48	58.39	57.39	57.93	3.03
**Bout1_3**	57.68	56.44	52.25	52.61	52.28	50.25	52.44	2.02
**Bout1_4**	63.98	61.58	59.80	60.18	62.72	64.06	61.95	1.59
**Bout1_5**	39.40	37.66	37.63	38.51	37.97	37.95	33.75	1.74
**Bout1_6**	48.19	46.63	39.17	39.46	43.80	43.85	45.57	3.11
**Bout1_7**	42.15	40.18	37.21	37.19	38.08	38.81	38.62	1.13
**Bout1_8**	39.57	39.32	36.79	37.77	36.11	38.66	36.84	1.23
**Bout2_1**	61.40	59.96	59.79	58.72	58.88	61.04	60.30	0.87
**Bout2_2**	70.92	70.37	69.66	66.27	69.00	67.82	66.81	1.62
**Bout2_3**	63.28	61.53	58.60	58.70	59.60	58.45	58.40	1.22
**Bout2_4**	68.66	68.16	65.73	66.86	63.97	59.82	65.14	2.89
**Bout2_5**	42.00	41.37	40.78	40.69	42.56	40.82	41.59	0.71
**Bout2_6**	49.42	50.07	45.89	44.75	47.49	47.11	45.30	1.92
**Bout2_7**	46.59	46.46	45.94	47.08	47.11	63.42	51.15	6.74
**Bout2_8**	47.26	48.68	45.89	44.17	46.67	43.42	46.47	1.89
**Average Difference against GT3X**		1.19 (2.25%)	2.75 (5.27%)	1.15 (2.22%)	1.57 (3.29%)	2.42 (4.90%)	2.41(4.62%)	2.03

**Table 5:** Pairwise correlation for the second experimental series.

	GT3X	Nexus 6	Pixel-1	Pixel-2	Pixel-3	Samsung	ZTE
**GT3X**	1.00	1.00	0.96	0.98	0.98	0.85	0.97
**Nexus 6**	1.00	1.00	0.97	0.98	0.99	0.86	0.97
**Pixel-1**	0.96	0.97	1.00	0.99	0.98	0.88	0.96
**Pixel-2**	0.98	0.98	0.99	1.00	0.98	0.89	0.97
**Pixel-3**	0.98	0.99	0.98	0.98	1.00	0.90	0.98
**Samsung**	0.85	0.86	0.88	0.89	0.90	1.00	0.93
**ZTE**	0.97	0.97	0.96	0.97	0.98	0.93	1.00

## Data Availability

Raw and processed data can be downloaded from https://cs2.uco.edu/~fu/research/Sensor/ (accessed on 20 December 21).
